# Evolution of cooperation under indirect reciprocity and arbitrary exploration rates

**DOI:** 10.1038/srep37517

**Published:** 2016-11-28

**Authors:** Fernando P. Santos, Jorge M. Pacheco, Francisco C. Santos

**Affiliations:** 1 INESC-ID and Instituto Superior Técnico, Universidade de Lisboa, IST-Taguspark, 2744-016 Porto Salvo, Portugal; 2 ATP-group, P-2744-016 Porto Salvo, Portugal; 3 Centro de Biologia Molecular e Ambiental, Universidade do Minho, 4710 - 057 Braga, Portugal; 4 Departamento de Matemática e Aplicações, Universidade do Minho, 4710 - 057 Braga, Portugal

## Abstract

Cooperation has been recognized as an evolutionary puzzle since Darwin, and remains identified as one of the biggest challenges of the XXIst century. Indirect Reciprocity (IR), a key mechanism that humans employ to cooperate with each other, establishes that individual behaviour depends on reputations, which in turn evolve depending on social norms that classify behaviours as good or bad. While it is well known that different social norms give rise to distinct cooperation levels, it remains unclear how the performance of each norm is influenced by the random exploration of new behaviours, often a key component of social dynamics where a plethora of stimuli may compel individuals to deviate from pre-defined behaviours. Here we study, for the first time, the impact of varying degrees of exploration rates – the likelihood of spontaneously adopting another strategy, akin to a mutation probability in evolutionary dynamics – in the emergence of cooperation under IR. We show that high exploration rates may either improve or harm cooperation, depending on the underlying social norm at work. Regarding some of the most popular social norms studied to date, we find that cooperation under *Simple-standing* and *Image-score* is enhanced by high exploration rates, whereas the opposite occurs for *Stern-judging* and *Shunning*.

The act of cooperation is generally framed quantitatively as an interaction in which an individual provides a benefit *b* to another at a cost *c* to himself^1^,^2^,^3^. Traditionally, one assumes that the benefit exceeds the cost (*b* > *c*). This means that whenever two individuals are given the option to cooperate or not (that is, to defect) with each other, the joint social optimum is achieved when both cooperate; yet, for each one individually, the fact that cooperation is costly configures defection as the preferred option^4^. In this sense, cooperation embodies a fascinating social dilemma within societies. When played bilaterally and simultaneously, this donation game turns into a Prisoner’s Dilemma (PD), which provides a convenient abstraction of a wide range of human interactions where the pursuit of self-interest leads to poor collective outcomes. In this context, two fundamental questions have puzzled scientists since Darwin: How is cooperation so widespread in human societies? How can cooperation emerge where it is absent?

The human capacity to establish and use reputation systems suggests some answers. Indeed, humans developed a huge machinery used at profit to share information about others^5^,^6^; they evolved to shape decision-making based on the reputation of those they interact with; and they act influenced by what they want others to know about them. All together, reputations work as a mechanism of social control and a lever for cooperation, altruism and collective action^7^,^8^,^9^,^10^.

Cooperation and reputation have been mathematically linked in models of Indirect Reciprocity (IR)^11^,^12^,^13^,^14^,^15^,^16^,^17^,^18^,^19^,^20^,^21^,^22^,^23^,^24^,^25^,^26^,^27^,^28^,^29^,^30^,^31^,^32^,^33^,^34^,^35^,^36^,^37^,^38^,^39^,^40^,^41^,^42^. IR models comprise individuals who adopt heuristics for decision-making based on reputations. In general, the complexity of IR models is limitless. Let us consider the simplest scenario where reputations can either be good (G) or bad (B), and individuals can adopt one of two possible actions: to cooperate (C) or to defect (D). As before, if they cooperate they loose *c* and the opponent earns *b*. Otherwise, they pay no cost and confer no benefit. The decision to opt between C and D is not arbitrary. It is encoded in an action rule that prescribes what to do against a G or a B opponent. Naturally, four strategies emerge: always cooperate (AllC); always defect (AllD); discriminate between G and B, only cooperating with G (Disc); or cooperate uniquely with a B (pDisc). Here we assume that the adoption of each strategy follows a process of social learning^43^ i.e., at each time step, one individual is picked and is given the opportunity to imitate the strategy of a model agent depending on its (better) performance.

Within this environment, reputations are dynamic. After each interaction, the reputation of the individual who decided between C or D (called the donor) when facing another individual (called the recipient) will eventually change. The rule that settles the new reputation to the donor given his actions and the characteristics of himself and the recipient is commonly called a social norm^14^,^20^,^21^. The present work involves the so-called 2^nd^-order social norms^21^,^23^,^31^. Mathematically, such a norm can be represented as a vector of the type [*w*, *x*, *y*, *z*], where each position provides the information regarding the new reputation of the donor (G/B), given the reputation of the recipient (G/B) and the action of the donor (C/D): *w* is the new reputation of an individual that chose C when facing a G opponent; *x* is the new reputation of an individual that chose D when facing a G opponent; *y* is the new reputation of whoever chose C when facing a B opponent; and finally *z* is the new reputation of an individual that opted for D when facing a B opponent. This implies the existence of 16 social norms. Out of these, taking symmetries into account (G and B can be swapped^19^ as, *a priori*, they have no meaning and thus constitute pure labels), only 10 social norms are truly distinct. For similar pairs of norms, we discuss the norm that attributes a positive valuation to G, given its relation with C. Popular examples studied in the past are^18^,^21^,^31^: Image-Score (IS) [G, B, G, B]^16^,^17^; Simple-Standing (SS) [G, B, G, G]^23^,^35^,^44^; Shunning (SH) [G, B, B, B]^28^,^45^; Stern-Judging (SJ) or Kandori [G, B, B, G]^30^. Interestingly, each social norm defines the dynamics of reputation assessment, which in turn impacts the payoff obtained by each behavioural strategy and consequently their representation in the population. This very simple setting defines a model of IR (detailed in Methods) whose fingerprint is present in a vast set of IR works^13^,^16^,^19^,^20^,^21^,^22^,^31^. In Fig. 1, in order to provide an intuitive visualisation, we use a ring construction^30^ to depict some of the most popular social norms studied to date.

So far, IR models neglected the strategic ambiguity that characterizes human interactions by emphasizing strategy adoption through fitness driven mechanisms only (e.g., social learning, cultural imitation, genetic inheritance, etc.) and disregarding the spontaneous adoption of new behaviours. This assumption is questionable, however. The emotional nature of many processes of individual decision-making, together with the creative urge to try new behaviours and the inability to assess accurately the reputation or success of others, all add up to augment the ambiguity associated with the process of strategy adoption. This behavioural ambiguity has been modelled employing a changeable exploration rate, that is, a varying probability that a new strategy is adopted without any sort of individual or social influence^46^,^47^,^48^. This process interestingly resembles (and may be formulated in a mathematically identical way) biological mutations in genetic settings. However, whereas in genetics random mutations are often rare, in social evolution this is not necessarily the case^46^: On the contrary, high exploration rates may turn out to be the norm rather than the exception, which may strongly affect the evolutionary dynamics of populations facing cooperation^47^,^49^ and fairness dilemmas^50^. In this work we address this issue in the context of IR. We resort to the toolkit of evolutionary game theory (EGT)^43^ and numerically explore the dynamics of strategy adoption, when social norms govern the co-evolving dynamics of reputation assignment and when individuals may spontaneously adopt (with arbitrary exploration rates) any strategy^47^. We compute the so-called stationary distribution of strategies in finite populations of size Z, and the population-wide gradients of selection, which allow us to characterize, in detail, the dynamics of strategy adoption. We find that the strategy ambiguity stemming from high exploration rates favours cooperation under the norms *Image-Score* (**IS**) and *Simple-Standing* (**SS**), whereas it inhibits cooperation under *Shunning* (**SH**) and *Stern-Judging* (**SJ**).

## Results

**Figure 1:**
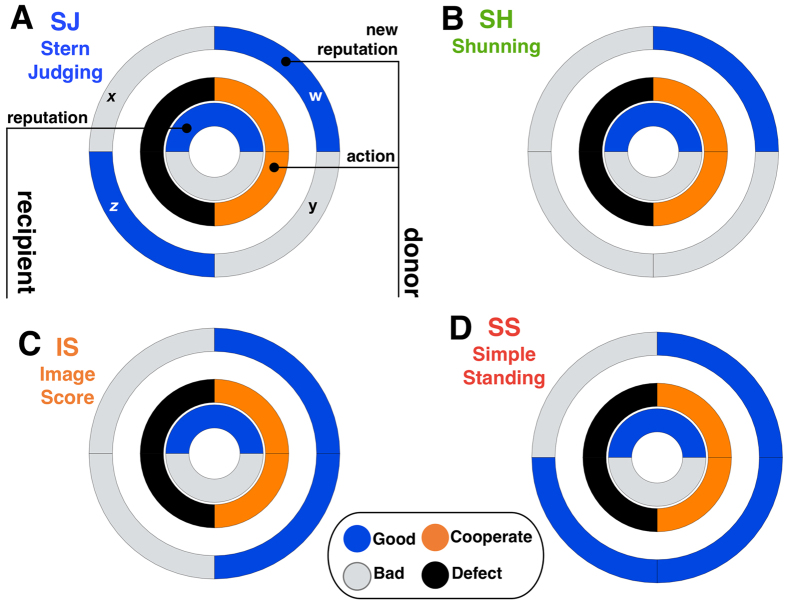
Ring representation of 4 of the most popular social norms. A social norm can be thought of as a vector of the type [*w*, *x*, *y*, *z*]. Each position provides the new reputation of the donor and, in a ring illustration, this information is placed in the outer ring with G = Blue and B = Grey (panel **A**). This new reputation depends on the reputation of the recipient (innermost ring with G = Blue and B = Grey) and on the action of the donor (middle ring, where C = Orange and D = Black). There are 16 possible social norms combining all these *bits of information*. 4 of the most popular are (**A)** Stern-judging: [G, B, B, G]; (**B**) Shunning: [G, B, B, B]; (**C**) Image-score: [G, B, G, B] and (**D**) Simple-standing: [G, B, G, G].

**Figure 2:**
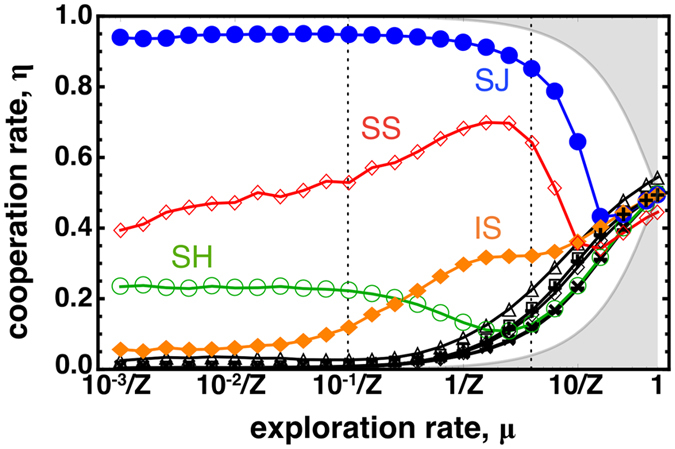
Effect of exploration rate in the cooperation sustained by each social norm. Cooperation under Simple-standing (**SS**) and Image-score (**IS**) profits from high exploration rates (μ); conversely, cooperation rates under shunning (**SH**) and Stern-judging (**SJ**) decrease with high μ (see Fig. 1 for definitions of these social norms). The other social norms considered are unable to promote cooperation under a wide range of μ (black lines). When μ approaches 1, individuals pick strategies randomly and social learning has no effect in the overall evolution of decision making; hence cooperation increases compared with the reference scenario. Furthermore, for high μ there is a configuration subspace that becomes inaccessible (i.e., μ implies a minimum prevalence of each strategy), which imposes limits to η. These limits are represented with a grey background. Dashed lines represent the exploration rates studied in detail in Figs 3 and 4. Other parameters (see section Methods): Z = 50, *b* = 5, *c* = 1, χ = ε = α = 0.01, τ = 1.

In Fig. 2 we depict, for a wide interval of exploration rates (from 10^−3^/Z to 1) and Z = 50, the cooperation levels associated with each social norm. We use specific colours to represent the behaviour associated with the 4 most popular 2^nd^-order social norms, defined in Fig. 1. As shown, whenever μ > 10^−1^/Z (indicated by the leftmost vertical dashed line) the cooperation level associated with each social norm is clearly affected by high exploration rates, with the ranking of each norm even changing. While **SJ** and **SS** preserve the status of norms that noticeably sustain more cooperation, there are important effects worth pointing for large values of μ: (1) **IS** and **SS** benefit, in most cases, from higher exploration rates; (2) the cooperation rate under **SH** and **SJ** slightly decreases for high exploration rates and (3) as μ approaches 1, cooperation in all the remaining social norms (drawn with a black colour) generally increases with μ, while cooperation rates under all norms approach 0.5.

Let us start by clarifying point 3): Increasing the exploration rate implies that social learning plays a decreasing role in the overall evolution of decision making. In particular, for very large mutation rates, since we have a significant fraction of the population (μ) just exploring the strategy space, the system typically does not access the grey shaded areas pictured in Fig. 2 (see Methods for details). This way, those social norms that are unable to promote cooperation under social learning benefit from the co-existence of all strategies. Naturally, the converse happens for those norms that already amplify the levels of cooperation under a regime of social learning, as is the case of **SJ** and **SS**. In the following discussion, we focus on points (1) and (2), i.e., on the interval of μ in which fitness dependent social learning — and thereby reputations and social norms —play the steering role in strategy adoption.

**Figure 3:**
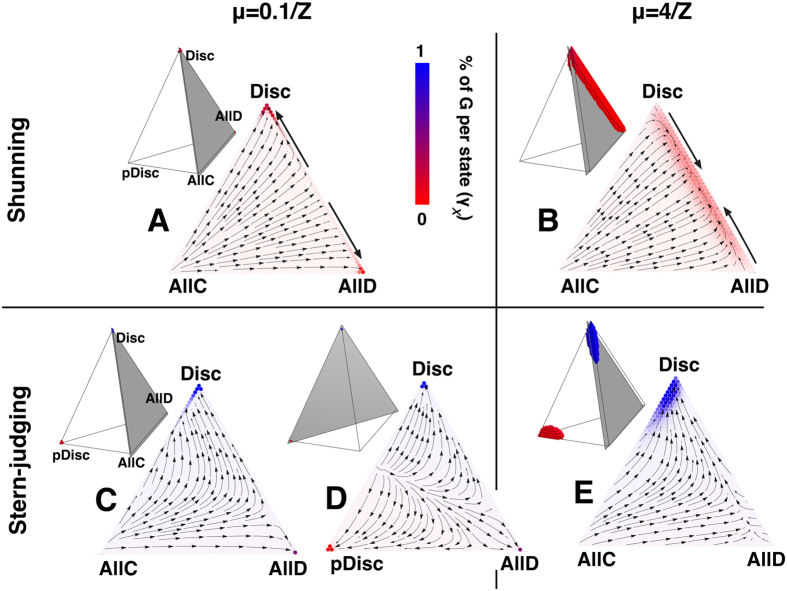
Dynamics of strategy adoption under Shunning (SH) and Stern-Judging (SJ). Tetrahedrons represent the full state space (see section Methods), in which a tiny sphere (coloured given the ratio G/B) is placed in the configuration states where the population spends more time (*λ*_*x*_), until 80% of total simulation time is covered. For convenience, the gradient of selection is visualized in the cross sections (triangles) whose location in the tetrahedron is indicated with a grey shade. Arrows represent the gradient of selection (*Γ*_*x*_), i.e., the most likely trajectory (in configuration space) that the population will follow once at given state. The colour of the spheres (tetrahedron) and circles (triangles) reflects the blend *γ*_*x*_ Blue + (1 − *γ*_*x*_)Red shown (where *γ*_*x*_ gives the fraction of Good individuals in each state). Both in **SH** (top panels, triangles A and B) and **SJ** (bottom panels, triangles **C**–**E**), high values of μ (right panels) slightly decrease the cooperation rate, compared to lower values of μ (left panels). Under low μ (triangles **A**,**C**,**D**) most of the time is spent in monomorphic states that promote cooperation (Disc for **SH** and **SJ** and also pDisc for **SJ**). Under high exploration rates (triangles **B**,**E**) the population is pushed away from these states, in which case cooperation is slightly affected (see Fig. 1). Other parameters (see section Methods): *Z* = 50, *b* = 5, *c* = 1, χ = ε = α = 0.01, τ = 1.

**Figure 4:**
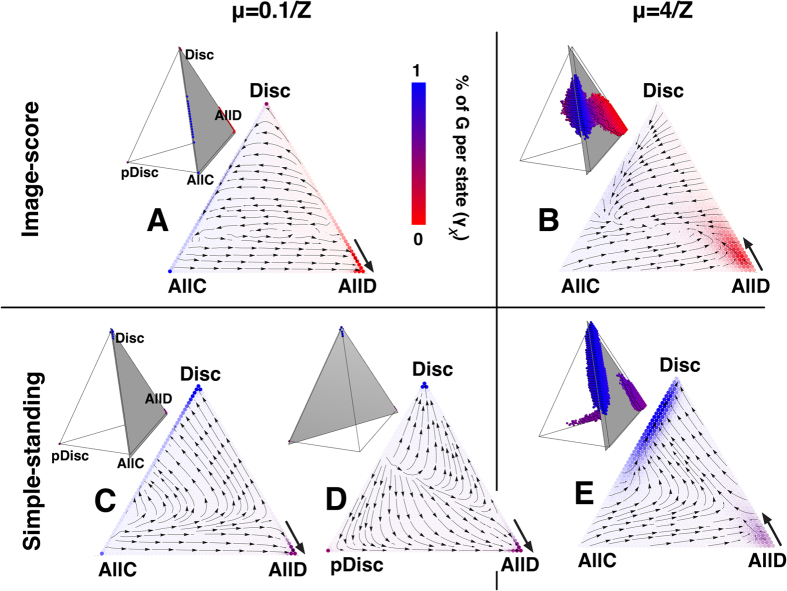
Dynamics of strategy adoption under Image-score (IS) and Simple-standing (SS). We use the same notation as in Fig. 3. Both under **IS** (top panels, triangles **A**,**B**) and **SS** (bottom panels, triangles **C**–**E**), the cooperation rate increases for high μ (right panels, triangles (**B**,**E**), see also Fig. 1). For low μ (left panels, triangles **A**,**C**,**D**), a lot of time is spent near the monomorphic state AllD, where cooperation is absent. For high μ AllD looses stability and the population is pushed to the interior of the simplex, where it is easier to fall into configuration states with a significant number of Disc (**SS**, triangle **E**) or to incur into a cycling dynamics in the interior of the simplex (**IS**, triangle **B**). In both cases, more individuals cooperate. Other parameters (see section Methods): *Z* = 50, *b* = 5, *c* = 1, χ = ε = α = 0.01, τ = 1.

To clarify the points 1) and 2) and to further understand the effects of high exploration rates on norms **SH**, **SJ**, **IS**, and **SS**, we show in Figs 3 and 4 *i*) the gradients of selection (*Γ*_*x*_), together with *ii*) the reputation distribution per state (*γ*_*x*_) and *iii*) the stationary distribution (*λ*_*x*_) of strategies, for the 4 most popular social norms (see section Methods for a detailed account of these metrics). The tetrahedrons represent the entire state space (simplex) defined by this 4-strategy dynamics, where each corner defines a monomorphic state in which the entire population adopts the same strategy. For simplicity and visualization purposes we provide details of the evolutionary dynamics inside the triangular slices of the complete 3D dynamics. Inside these triangular slices, each arrow corresponds to *Γ*_*x*_ and represents the most probable direction of evolution. The prevalence in each state (*λ*_*x*_) is associated with the background colour intensity whereas blue/red tones translate into an increased number of G/B individuals (*γ*_*x*_).

Figure 3 shows that the harshness of **SH** becomes more evident for high μ (Fig. 3-B), and cooperation is thereby precluded. High μ means that B labels are even easier to be attributed due to the presence of individuals that spontaneously adopt AllD and pDisc, strategies that in general are labelled B under **SH**. The increase in B individuals makes Disc and AllD almost indistinguishable and turns the coordination between these strategies (Fig. 3-A) into a co-existence dynamics (Fig. 3-B). In the case of **SJ** we observe that, for low μ, most of the time is spent in the highly cooperative monomorphic states Disc and pDisc (Fig. 3-C,D). When μ increases (Fig. 3-E) cooperation declines as the prevalence under monomorphic states Disc/pDisc decreases, with individuals exploring the other strategies (AllD and AllC).

Figure 4 reveals why cooperation increases at high μ, when the prevailing norm is either **IS** or **SS**. For **IS**, high exploration rates have the ability to move the system away from states where AllD is the prevalent strategy (Fig. 4-B). Indeed, high values of μ increase the number of individuals that spontaneously adopt Disc or AllC, placing and stabilizing the dynamics in the interior of the simplex. In the case of **SS**, high values of μ allow the population to overcome the “coordination barrier” between AllD and Disc (Fig. 4-E), thus making it easier to achieve the minimum number of Discs that renders advantageous to have a G reputation; this way, the population spends less time in AllD and more time near the edge where AllC and Disc co-exist, which naturally benefits cooperation.

Besides the particular effect of μ in each social norm, it is noteworthy that cooperation levels remain qualitatively unchanged (apart from the slow monotonic increase of cooperation under **SS**) for a wide range of values of the exploration rate μ (Fig. 2). These results confirm, for the first time, that for a wide interval of values of μ the Small-Mutation Approximation^31^,^51^ (SMA) proves accurate in the context of IR. With SMA, one assumes that μ is small enough so that the system will spent a negligible fraction of time in polymorphic states. This way, SMA allows a convenient characterization of evolutionary processes, formally in the limit when μ ≪ 1/Z, through a reduced (embedded) Markov chain involving monomorphic states only. Yet, its range of applicability is often unclear^48^, requiring the use of large-scale computer simulations as the ones performed here.

## Discussion

In this work we investigate the effect of an arbitrary exploration rate on the evolutionary dynamics of cooperation under indirect reciprocity. This is an analysis of general interest given the ubiquity of creative and emotional traits that characterize human behaviour, urging them to explore new strategies^47^. The reputation systems studied here were based on the action of the donor and the reputation of the recipient, a feature associated with so-called 2^nd^-order norms^21^,^31^. We show that random exploration of the strategy space has a non-trivial effect in the behaviour dynamics of populations under IR: when high, it increases cooperation under **IS** (interestingly, a norm just demanding 1^st^ order information) and **SS**, while it decreases cooperation under **SH** and **SJ**. Despite these key observations, our results indicate that the so-called “*leading two”* norms^21^ — **SJ** and **SS** — remain the most effective in promoting cooperation, a result that may not hold in the space of high-order norms. Overall, our results suggest that a general heuristics can, in fact, be intuitively derived: when the social norm is able to provide high cooperation rates by relying on the stability of some cooperative monomorphic states (e.g., Disc in **SJ** and **SH**), exploration is pernicious; when the social norm is unable to provide that stability, allowing instead for the occurrence of some uncooperative monomorphic state (e.g., AllD in **IS** and in **SS**) high exploration rates favour overall cooperation by allowing the population to move away from this undesirable state.

Despite these results, we confirm that **SJ** remains the social norm that, overall, promotes the highest levels of cooperation. The strength of this norm relies on the efficiency to promote a strong stability of two highly cooperative monomorphic states. No other polymorphic state (i.e., in which more than one strategy co-exists) is able to reach those levels of cooperation; hence, any ambiguity source that moves the population to the interior of the simplex will be detrimental to cooperation. This, however, is not the whole story, since the reference levels of cooperation attained under **SJ** are so high that the disadvantageous effect induced by large exploration rates does not prevent **SJ** to remain the leading norm in what concerns the promotion of cooperation.

The dynamics under **SJ** also puts in evidence a remarkable fact. Because of the inherent symmetry of **SJ** with respect to the G and B labels, the net effect of **SJ** resembles some sort of a divide-and-conquer procedure. Indeed, under **SJ** the whole state space is divided into two smaller basins of attraction that push the population to full cooperation in both cases: if there is a majority of individuals that cooperate with B and defect with G (pDisc), the population will move into a state where everyone is B; if there is a majority of Disc, the population will move to a state where everyone is G; in both limits, cooperation prevails, and that ensures the collective optimum (Fig. 3-D). Ultimately, the notions of Good and Bad, as we normally use them, are defined by the actions and not by the labels attributed to specific reputations. Clearly, in the case of **SJ**, the meaning of the *signals* G(ood) or B(ad) may emerge from a simple convention^52^,^53^. This means, in turn, that if we would consider different populations evolving independently and under the assessment of this norm, full cooperation might still be achieved under different emergent conventions for what is Good or Bad. Given the conceptual simplicity of the underlying IR model employed, this constitutes a remarkable feature of **SJ**, related also to complex topics such as conflicting moral systems^30^,^54^ or the appearance of in/out groups together with their inherent normative values. These observations can only be made, however, to the extent that (like we have done in this work), one provides the global dynamics considering the 4 possible action rules (AllD, Disc, pDisc, AllC), instead of carrying out the analysis including only 3, as often happens. This feature, together with the systematic investigation of high exploration rates in IR, were carried out here for the first time.

It is also noteworthy the way **SJ** implies a moral judgement that, besides justifying the defection against undesirable opponents, also condemns cooperating with those. Indeed, under **SJ**, whoever cooperates with an opponent carrying a Bad reputation gets himself a Bad reputation. Interestingly, this judgement is somehow verified in the behaviour of toddlers, who prefer those that mistreat (rather than help) opponents that misbehaved in the past^55^,^56^,^57^,^58^.

Finally, given the current importance of reputation-based systems, indirect reciprocity models emerge nowadays as relevant toolkits for artificial intelligence applications, web platforms and systems supporting *sharing economies*. Online communities are nowadays pervasive and most of them profit from the readiness of its users to cooperate, which is often supported by reputation^59^. Overall, reputation mechanisms are considered a key element in the design of multiagent systems^60^,^61^,^62^. The simulation technique that we present here, together with the proposed metrics to visualize the emergent dynamics, can provide the appropriate basis in which to study (and evaluate ref. 63) other challenges related with strategic dynamics and reputation systems, such as the effect of considering different reputation management schemes^64^,^65^,^66^,^67^, the design of new underlying structures of interaction^36^,^68^,^69^,^70^,^71^,^72^,^73^,^74^ or the formalization of bottom-up artificial morality and machine ethics^75^.

## Methods

### Strategies and reputations

We model a population of *Z* individuals that interact with each other and may change their strategy over time (in the way described below). Individuals play with each other a donation game that reproduces, in a simple way, the mathematics of cooperation^43^. Each individual may choose one of two actions: to cooperate (C), meaning that the donor incurs a cost *c* to confer a benefit *b* to the opponent; to defect (D), whereby neither player earns or looses anything. Each individual follows a strategy (action rule) that defines how to act against a Good (G) or Bad (B) opponent. This rule is encoded in a vector [x, y] where the first position dictates what to do (C/D) against a G and the second position what to do against a B. There are four possible strategies: AllC [C, C], Disc [C, D], pDisc [D, C] and AllD [D, D]. Initially, each individual has one reputation (G or B) and strategy; these are attributed randomly with uniform probability.

### Update of strategies and reputations

At each (discrete) time step (*t*) one individual X is randomly selected to update its strategy. With probability μ (so called exploration rate^43^,^46^,^47^,^48^) X adopts a random strategy within the full space of possible strategies. With probability 1**-μ strategy change may take place through social learning; in this case, X compares its fitness (*f*_*X*_) with that (*f*_*Y*_) of another individual Y randomly selected, changing its strategy to that of Y with a probability that increases with the fitness difference, given by 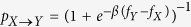, with β = 1, ensuring a significant selection strength. This imitation process and the associated probability function are well documented^47^,^76^ and known as pairwise comparison rule. Both fitnesses *f*_*X*_ and *f*_*Y*_ are associated with the average payoff obtained in 2*Z* donation games, always played against random opponents. In each of these games, both individuals play once as donor and once as recipient. After each donation game, with a probability *τ*, a new reputation is attributed to the individual acting as donor, in accordance with the social norm fixed in the population. With probability 1 − *τ*, the donor keeps the same reputation. For simplicity, we assume a public reputations scheme^16^,^19^,^20^,^31^ such that, through gossip or rumours, new reputations spread to everyone, a simplification that can naturally be relaxed in future works based on different reputation database schemes^64^. As already described in the Introduction and depicted in Fig. 1, here we consider that a social norm is a vector of the type [*w*, *x*, *y*, *z*], where each position provides the information regarding the new reputation of the donor (G/B), given the reputation of the recipient (G/B) and the action of the donor (C/D).

### Errors

We allow for the inclusion of errors of three different types: execution errors – with probability *ε*, there is a failure to cooperate when the action rule dictates so^34^; assignment errors – with probability *α*, the assigned reputation is the opposite of the one prescribed by the social norm; and private assessment errors – with probability *χ*, when deciding about what action to employ or when deciding the next reputation of an opponent, the retrieved reputation of an individual is the opposite of the one actually owned^21^. The effect of each kind of error in the cooperation levels supported by each specific social norm was already discussed^31^. While Figs 1, 2 and 3 report the results for *ε* = *α* = *χ* = 0.01, we confirm that the effect of exploration rate discussed in the main text remains unchanged if (all else constant) *ε*, *α* or *χ* go up to 0.1 and *τ* down to 0.01.

### Tracing the dynamics

Each possible state of this population can be enumerated and arranged spatially, so that its dynamics becomes straightforward to visualize. As there are *Z* individuals and 4 different strategies, each state of the population is identified by a tuple *x* = (*k*_*0*_, *k*_*1*_, *k*_*2*_) meaning that, in state *x*, there are *k*_*0*_ individuals adopting strategy AllD, *k*_*1*_ adopting strategy pDisc, *k*_*2*_ adopting strategy Disc and *k*_3_ = Z*-k*_*0*_*-k*_*1*_*-k*_*2*_ adopting strategy AllC. In total, there are (Z + 1) (Z + 2) (Z + 3)/6 states. These states can intuitively be assembled in a 3D simplex (a tetrahedron as in Figs 3 and 4), where the vertices represent states in which all the agents are adopting the same one unique strategy (monomorphic or pure states). In Figs 3 and 4, to allow for an easier interpretation of the results, we present the dynamics along cross sections of this space (2D simplexes, triangles of variable size depending on the specific cross section position). Provided this description, we retrieve information in four different forms discussed below: average cooperation rates, average time in each state, average fraction of good and bad reputations, and gradient of selection.

### Average cooperation rate

We use the general result that one generation corresponds to Z discrete time steps where a strategy update may occur, and we simulate the system for G = 10^7^ generations. We repeat each simulation R = 100 times (runs), such that in each run the pseudo-random number sequence will be different. The average cooperation rate (*η*_*i*_) in run *i* is computed by dividing the total number of cooperative acts (C_i_) by the total number of donation games (K_i_):


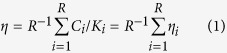


As μ → 1, exploration increasingly dominates selection (in the form of social learning), implying that reputations and social norms are subjected to minor perturbations with respect to a purely random choice process. Particularly, since there is always the probability μ that an individual explores any other strategy, the space of accessible configurations is effectively reduced with the increase of μ^47^, such that 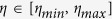. The minimum fraction 
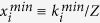
 of strategy *i* is given by one of the roots of 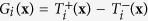, where 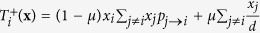 and 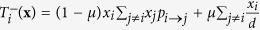 are the probabilities of increasing and decreasing (respectively) the number of individuals of strategy *i*, for a general state **x**. In the most unfavourable scenario for strategy *i* (i.e., strong selection (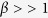), 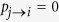 and 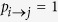), 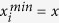 can be calculated by solving 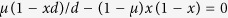, which results in 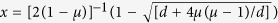. This quantity is close to *μ*/*d*, as employed in ref. 47. In general *η* = *k*_*3*_/Z + γ*k*_*2*_/Z + (1 − γ)*k*_*1*_/*Z* - with γ standing for the fraction of G individuals (see below) — which is minimized for *k*_*1*_/*Z* = *k*_*2*_/*Z* = *k*_*3*_/*Z* = *x*, such that, for *d* = 4, *η*_*min*_ = *x* + γ*x* + (1 − γ)*x* = 2*x*. A similar argument can be used to compute the minimum defection rate, leading to a *η*_*max*_ = 1 − *η*_*min*_. The inaccessible cooperation rates 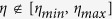 are represented in Fig. 1 by a grey background.

### Average time in each state

We keep a counter that totalizes the number of time steps a given state of the population is reached. After the proper normalization (the total number of steps is GRZ), we collect information on the average fraction of time (*λ*_*x*_) that the population spends in each state *x* = (*k*_*0*_, *k*_*1*_, *k*_*2*_). This information is conveyed in the triangles of Figs 3 and 4 by means of colour intensity. In the tetrahedrons, we place a sphere in the states where the population spends more time. The area covered by the spheres accounts for 80% of the total simulation time, i.e., assuming that state *x*_*0*_ is the most visited state, 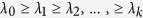, we place spheres up to state 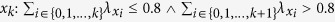.

### Average fraction of Good and Bad reputations

By directly placing the system in each possible state *x* = (*k*_*0*_, *k*_*1*_, *k*_*2*_) and simulating one time step per run (total number of runs R, each starting in the same state *x*), we can draw a picture of the average number of G/B. After each time step we save the resulting fraction of G individuals, G_*t*,*x*_/(G_*t*,*x*_ + B_*t*,*x*_). After all runs, we have the average fraction of G individuals accruing from a specific state *x*,


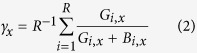


### Gradient of selection

Following a similar procedure as the one described in the previous section, we can compute the transition probability between each pair of neighbour states *x* and *y*. For each state *x* = (*k*_*0*_, *k*_*1*_, *k*_*2*_), we count the number of times that, after each time step, the system moves into each of the (maximum) 12 adjacent states *y* = (*l*_*0*_, *l*_*1*_, *l*_*2*_). For that, we keep the quantities *β*_*x*,i_ and *δ*_*x*,*i*_, i.e., the number of times that a strategy *i* is born (*l*_i_ = *k*_*i*_ + 1) or dies (*l*_i_ = *k*_*i*_ − 1) in state *x*. We keep the number of runs that we start a time step in each state in *x*, R_*x*_. This provides the required information to represent the so-called gradient of selection *Γ*_x_, i.e., a vector field that provides an approximation for the most probable direction that the system will follow once located in state *x*, given the update of strategies (imitation and exploration) by each agent (described above),


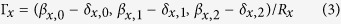


## Additional Information

**How to cite this article**: Santos, F. P. *et al*. Evolution of cooperation under indirect reciprocity and arbitrary exploration rates. *Sci. Rep*. **6**, 37517; doi: 10.1038/srep37517 (2016).

**Publisher’s note:** Springer Nature remains neutral with regard to jurisdictional claims in published maps and institutional affiliations.
